# Building knowledge translation competency in a community-based hospital: a practice-informed curriculum for healthcare providers, researchers, and leadership

**DOI:** 10.1186/s13012-020-01013-y

**Published:** 2020-07-03

**Authors:** Christine Provvidenza, Ashleigh Townley, Joanne Wincentak, Sean Peacocke, Shauna Kingsnorth

**Affiliations:** 1 Holland Bloorview Kids Rehabilitation Hospital, 150 Kilgour Road, Toronto, ON M4G 1R8 Canada; 2 Bloorview Research Institute, Holland Bloorview Kids Rehabilitation Hospital, Department of Occupational Science & Occupational Therapy, Rehabilitation Sciences Institute, University of Toronto, 150 Kilgour Road, Toronto, ON M4G 1R8 Canada

**Keywords:** Knowledge translation, Competency, Training, Curriculum development

## Abstract

**Background:**

Enacting knowledge translation (KT) in healthcare settings is a complex process that requires organizational facilitation. In addition to addressing organizational-level barriers, targeting individual-level factors such as KT competencies are a necessary component of this aim. While literature on KT competency training is rapidly growing, there has been little exploration of the potential benefits of training initiatives delivered from an intra-organizational perspective. Addressing this gap, we developed the Knowledge Translation Facilitator Network (KTFN) to meet the KT needs of individuals expected to use and produce knowledge (e.g., healthcare providers, research staff, managers, family advisors) within an academic health sciences center. The aim of this study is to describe the development, implementation, and evaluation of the KTFN curriculum.

**Methods:**

An educational framework was used to guide creation of the KTFN curriculum*.* Stakeholder interviews, a literature review of KT competency, and environmental scan of capacity building initiatives plus adult learning principles were combined with in-house experience of KT practitioners to inform content and delivery. An evaluation strategy consisting of pre/post-test curriculum and post-session satisfaction surveys, as well as post-curriculum interviews assessed impact on participant knowledge and skills and captured perceived value of KFTN.

**Results:**

The curriculum has been delivered three times over 3 years, with 30 individuals trained, representing healthcare providers, graduate level research trainees, managers, and family advisors. Using the New World Kirkpatrick Model as an analysis framework, we found that the KTFN curriculum was highly valued and shifted learners’ perceptions of KT. Participants identified enhanced knowledge and skills that could be applied to different facets of their work; increased confidence in their ability to execute KT tasks; and intention to use the content in future projects. Barriers to future use included time to plan and conduct KT activities.

**Conclusion:**

KTFN was developed to enhance KT competency among organizational members. This initiative shows promise as a highly valued training program that meets both individual and organizational KT needs and speaks to the importance of investing in tailored KT competency initiatives as an essential building block to support moving evidence into practice.

Contributions to the literature

- Externally focused KT practice training initiatives have been established to build KT competencies among diverse knowledge users. An area for continued exploration is building internal KT capacity within individual healthcare settings to drive organizational initiatives.
- The KTFN is an exemplar of an organizational-driven, curriculum-based initiative to build KT competencies across a diverse group of multi-disciplinary individuals working collaboratively on strategic projects.
- Outcomes of this work highlight the importance of enhancing KT competencies, such as knowledge and practical skills, which have the potential to build organizational KT capacity and enhance impact in research, education and care.

## Background

Knowledge translation (KT) in healthcare is a dynamic and iterative process aimed to put evidence into practice [1]. This umbrella term encompasses a number of interrelated activities such as gathering, synthesizing, sharing, and implementing research evidence, with the ultimate goal of improving health-related outcomes at the patient and/or system level [2]. Effective KT practice requires the active participation and collaboration of individuals who research, plan, implement, adopt, and benefit from evidence-based healthcare interventions [3–5]. These “knowledge users” may include researchers, operations managers, professional practice leaders, healthcare providers, patients, and families. It is widely acknowledged that successfully enacting KT within healthcare is a complex and challenging process [5] and much effort is being devoted to understand how to build KT capacity.

One area of exploration to advance effective KT practice relates to KT competency development [1, 6–8]. Recent work by Mallidou and colleagues [9] identified and formally categorized 16 KT competencies for general practice within the structure of knowledge, skills, and attitude. Examples of KT competencies include understanding KT processes (knowledge), using strategies to share knowledge (skill), and valuing teamwork (attitudes). Aligned with an emphasis on building KT competencies, there has been a growth in curriculum-based initiatives to foster KT practice among knowledge users [9]. The target audiences of these training initiatives vary; some have focused exclusively on a group with a shared background, such as researchers [10] or discipline-specific knowledge users (e.g., occupational therapists [11] or primary care providers [6]). Other initiatives have capitalized on potential benefits of inter-professional learning and collaboration with mixed backgrounds recruited as individuals or teams across healthcare organizations [12, 13].

In a call to action, Holmes et al. [5] highlight the importance of organizational facilitation of KT, speaking to the need for healthcare organizations to build internal capacity to enact KT activities. Capacity building can involve establishing organizational structures and processes [14]. Embedding KT-specific roles (e.g., knowledge brokers, KT specialists) to facilitate staff (i.e., knowledge users) in KT activities is one approach gaining footing [4, 15]. However, even in organizations with devoted KT roles/teams, the number and scope of projects often far exceed the resources available for support; only select KT activities may be undertaken and/or complex KT projects prioritized. Effective KT practice, however, requires the active participation of all those responsible for producing and using knowledge. In addition to organizational structures, efforts to build KT capacity at the individual level are crucial [5, 14]. Increasing the ability of individuals within existing roles to do “better KT” can enhance delivery of evidence-based healthcare overall [5, 14].

### Context

Holland Bloorview Kids Rehabilitation Hospital is a large pediatric rehabilitative hospital for children with physical disabilities and complex needs in Toronto, Ontario. As an academic health sciences centre, the hospital recognizes the need to foster the integration of clinical care, research, and education in order to deliver evidence-based, high quality, and compassionate care in day-to-day practice [15, 16]. One key integration initiative aligned with this goal was development of the *Centres for Leadership*, a project-based funding program aimed at advancing collaborative and innovative solutions in clinical, research and education domains within the Hospital [17].

The Centres for Leadership program empowers multi-disciplinary teams of healthcare providers, researchers, graduate-level trainees, and family advisors to design, test, and implement solutions that will lead to a healthier future for children, youth, and families. To drive knowledge sharing and utilization, project teams are required to plan and execute KT activities with key audiences (typically families, healthcare providers, and researchers) as part of their peer-reviewed funding application. Historically, these KT plans often entailed narrow activities such as traditional peer-reviewed publications and presentations, focusing primarily on academic audiences. In cases where grander plans are proposed, the KT activities are often too ambitious or are pushed off beyond the funding timeframe (i.e., 12 months). Few applications leverage the opportunity to undertake or build a fulsome “KT” project focused on KT product development or planned implementation. There was thus a need for increased KT competency for individuals and teams to forecast additional phases of the work, apply KT frameworks or process models to inform projects, maximize integrated knowledge translation (iKT) principles, optimize awareness of outputs and findings with broad audiences, and produce theory-driven implementation and/or evaluation plans.

### Aim

To address this need, provision of a tailored curriculum to foster better understanding and integration of KT into projects, as well as empowering individuals to purposefully seek KT expertise and support was identified. We posit that opportunities to build foundational working knowledge of KT across an organization have the potential to strengthen KT competencies among diverse knowledge users. This aim was strongly endorsed by senior decision makers within the Centres for Leadership and the Hospital’s dedicated KT hub, *Evidence to Care* (EtC), with a mandate to accelerate evidence-informed decision making through KT practice [15]. A partnership was thus forged to create the Knowledge Translation Facilitator Network (herein called KTFN)*.* The term “facilitator network” was used purposely with a long-term vision of having a network of individuals with increased KT competency who could apply learnings to immediate Centres for Leadership projects, as well as other strategic hospital initiatives to generate impact. In this vein, a specialized KT curriculum focused on competency building was designed and evaluated to meet the needs of the organization’s knowledge users involved in integrated clinical, research and/or educational projects.

## Method

This paper reports on curriculum development and results of a mixed-method evaluation. Specific evaluative aims were to (i) explore satisfaction with and perceived value of the curriculum, (ii) assess the impact on KT competencies (knowledge, skills, and attitude), and (iii) determine confidence and intention of the learners to practice KT. Reporting adheres to guidelines provided in the Template for Intervention Description and Replication (TIDieR) [18]; a completed TIDieR checklist is available as a supplementary file.

### Evaluative design

A mixed-method, pre/post-test design was used for the first 3 years of delivery. The design was guided by The New World Kirkpatrick Model; a model suited to understanding if training programs deliver the “relevant knowledge and skills to the participants and the confidence to apply them on the job” ([19] , p. 5). This model comprised 4 levels. In the near and short-term, level 1: reaction addresses how favorably learners respond to the training; and level 2: learning focuses on the extent to which they acquire knowledge, skills, confidence, attitudes, and commitment. Level 3: behavior and level 4: results are focused on future behavior once they are back on the job and longer-term impact of their learning on organizational indicators, respectively [19]. As the evaluation was conducted during and immediately after KTFN curriculum delivery, evaluative activities focused on levels 1 and 2: reaction and learning. Appropriate to these levels, methodological triangulation tested the consistency of findings from three data sources: pre/post-test curriculum surveys, short post-curriculum interviews, and post-session satisfaction surveys [20].

### Participants

Participants in KTFN were selected based on actively leading strategic or integrated clinical, research, and/or education projects suitable for practical application and a declared professional interest in development of KT competency. No limits were placed on role; all staff including healthcare providers, senior management, students and trainees, research, and family advisors within these project teams were eligible to apply. Interested individuals submitted applications identifying their profession/role within the organization, their Centres for Leadership project, interest in KT competencies, and anticipated application of learnings. Annually, 10 to 15 participants were selected from the 18 to 20 annually funded project teams (average team size ranged from five to 15 members) to foster a small group learning environment.

### Curriculum resourcing

In addition to a Manager, Evidence to Care comprises a small team of KT practitioners (one × KT Specialist and two × Knowledge Brokers), all of whom are Masters’ trained in health related disciplines and hold advanced certification in KT practice. The KT Specialist led KTFN development, with additional support from the knowledge brokers around evaluation and session facilitation. Additional in-kind support was provided by the Centre for Leadership Manager, who was responsible for participant engagement and securing managerial approval including release time for clinical staff. Additional funding was provided annually to cover clinical release time, printing of materials, and session refreshments.

### Curriculum development and delivery structure

KTFN development was guided by Kern’s (2009) Six-Step Approach for Curriculum Development for Medical Education [21]. Designed for use by curriculum developers responsible for the educational experiences of healthcare professionals, this step-wise approach was an appropriate model given the target audience and delivery context [21]. A detailed account of the steps taken to create the curriculum are provided below.

#### Step 1: problem identification and general needs assessment

Problem identification has already been articulated earlier in this paper (see “Context” section). From a needs assessment perspective, the following aims were identified: (i) advance KT knowledge and skills across multi-disciplinary teams; (ii) instill value of KT and confidence to foster the integration of KT in clinical care, research, and education initiatives; and (iii) provide opportunities to practice applying KT knowledge and skills.

#### Step 2: targeted needs assessment of desired KT competencies

Information was collected to better understand KT competency needs within the organization. Senior decision makers in leadershiproles within the Centres for Leadership took part in brief interviews led by the KT Specialist to capture their perspectives, including one physician director, one physician/senior clinical scientist, one scientist, and one clinical director (*n* = 4). Collectively, they each had been Centre for Leadership Co-Leads and accountable for integration of clinical care, research and education for three or more years. Questions focused on perceived level of understanding of KT among project team members, types of dissemination, and implementation strategies currently being used and intended impact of the new KT curriculum. Gaps identified from these discussions included the lack of awareness and use of KT models, theories, and frameworks in KT planning; the need to use creative dissemination activities to enhance knowledge sharing; the importance of fostering stakeholder engagement (e.g., family advisors, external healthcare providers) in KT planning, product development, and other related activities; and the ability to conduct KT practices beyond dissemination, such as implementation and sustainability initiatives.

To compliment the interviews, a brief literature review of KT competencies, KT mentorship, and knowledge brokering activities was conducted and key findings important to KT practice identified [1, 22–25] and key findings important to KT practice identified. To further inform curriculum content, an updated environmental scan of readily available curriculum for KT practitioners, KT focused journals, and websites was also completed. An overview of the environmental scan is presented in Table 1.

**Table 1:** Overview of environmental scan

Type of document/resource	Purpose	Select Examples
Journals	Identify KT competency and role-based research and best practices (e.g., education curriculum peer-reviewed literature)	Implementation Science BMC Health Services Research BMC Medical Education
KT education programs	Review competencies identified by professional KT education programs designed for KT practitioners	Knowledge Translation Professional Certificate, Hospital for Sick Children Li Ka Shing Knowledge Institute, St. Michael’s Hospital
External KT agency websites	Scan public facing KT organizations	KT Canada National Collaborating Centre for Methods and Tools Center on Knowledge Translation for Disability and Rehabilitation Research

During the analysis and synthesis of the interviews, literature, and scan, curriculum developers discussed emergent learnings and further identified competency needs based on their own KT practice and experience in the organization.

#### Step 3: goals and objectives

The overall goals and objectives for the KTFN curriculum were informed by the interviews with the senior decision makers of the Centres for Leadership, external information gathering (literature and scan), and EtC’s contextual expertise of KT competencies required to enact KT. Bloom’s Taxonomy [26] was used as a framework to articulate sessional learning objectives, which focused specifically on the cognitive processes of building KT knowledge and fostering the comprehension and application of KT knowledge. Table 2 provides a description of sessional learning objectives.

**Table 2:** Focus and learning objectives of KTFN sessions

Session	Session 1^a^: Setting the stage for KT thinking	Session 2^a^: Organizing KT thinking	Session 3^a^: Putting KT thinking into motion	Session 4^a^: Putting KT planning into action: implementation and sustainability	Session 5^a^: Putting KT planning into action: Demonstrating impact	Session 6^a^: Putting KT planning into action: Applying KT knowledge
Sessional Learning Objectives (application of Bloom’s Taxonomy [26])	Describe KT Discuss the importance and value of integrating KT into the disability context	Identify core KT principles Distinguish between iKT and end-of-grant KT (eKT) Apply iKT and eKT frameworks to KT project plan	Discuss the benefits of KT planning Identify types of KT strategies Embed KT strategies into KT project plan to achieve goals	Identify different implementation strategies Identify sustainability constructs Embed implementation strategies and sustainability constructs into KT project plan	Identify and discuss KT indicators for goal achievement Identify collective hopes for impact for people in the system Embed evaluation strategies into KT project plan	Apply KT content learned through the delivery of a KT plan and participation in a simulation

iKT: stakeholders collaborate throughout the entire process to drive work (e.g., co-create objectives)

eKT: KT that happens at the end or completion of work (e.g. presentations, publications, products to prime for implementation)

^a^Reference to: KT terminology, seminal literature, KT theories, models, and frameworks was made throughout the sessions, where appropriate

#### Step 4: educational strategies

Curriculum delivery was group-based and delivered face-to-face. The KTFN learning environment was established as a collaborative space, wherein participants reflecting different disciplines were brought together to engage in active learning and discussion, critical-thinking, peer learning, reflection, and integration of new perspectives and beliefs related to KT. Table 3 provides a detailed list of the educational strategies informed by adult learning used. As part of curriculum delivery, participants received binders consisting of printed copies of lecture slides, reading lists of seminal papers, and worksheets.

**Table 3:** KTFN educational strategies

Strategy	Example execution
Didactic content	See Table 2
Think-Pair-Share [27]	Participants engaged in a think-pair-share activity to help them establish KT goals for their project. Participants were encouraged to think individually (think) about their KT goal(s). They engaged with a partner (pair and share) to share their goal(s) and acquire feedback. All participants engaged in a broader group discussion (share) to share ideas about their KT goals, foster their critical thinking about KT and maximize group participation.
Story-telling	Story telling was used by the EtC team and participants to share and reflect on lived experiences about KT. Attention was drawn to organizational facilitators and barriers to implementation of specific initiatives (e.g., highlight intra-organizational partnerships that can facilitate dissemination and implementation goals).
Case-studies	Pediatric rehabilitation-focused case studies were provided to the participants to foster their KT learning. Questions related to topics such as target audiences, dissemination strategies, and stakeholder engagement were posed to foster application of KT concepts.
Consultation	The EtC team provided consultative services to the participants. The participants were encouraged to seek assistance from the EtC team for items such as fostering their understanding of KT concepts or assistance with KT planning.
Independent KT planning	Participants engaged in independent KT planning where they applied concepts learned from KTFN to help them plan for their project. Planning involved identifying their KT goal, target audiences, key messages, stakeholder engagement opportunities and dissemination and implementation activities where appropriate.
Videos	Videos from websites such as the Ontario Centre of Excellence for Child and Youth Mental Health [28] were used to speak to the value of KT activities.
Guest lectures	Guest lecturers were both internal and external to the organization. Internal guest lecturers shared how they operationalized KT within the organization and discussed successes, pitfalls, and learnings. External guest lecturers provided in-depth knowledge on executing KT activities (e.g., evaluation).
Participant presentations	Participants presented their KT plan that was developed throughout the curriculum. These presentations were an opportunity for participants to showcase their work, gather feedback from their peers and demonstrate their application of KT concepts.
Simulation	Participants took on a persona/perspective unlike their current role, which helped them think through preconceived notions that all individuals bring to their work and the importance of considering stakeholder values and beliefs as part of the engagement process. How to have a conversation about KT: participants engaged with professional actors and focused on how to have a conversation about KT and the different elements and perspectives that need to be considered [29].

#### Step 5: implementation

Session delivery occurred in the spring and fall, with a summer break to accommodate scheduling needs of participants. Content was delivered across five to six sessions depending on modifications made annually based on prior cohort feedback. Facilitated by an EtC team member, each 3-h session was structured around three consistent components: (i) educational content delivered by KT practitioners, (ii) an expert guest lecturer in a related topic area, and (iii) active hands-on KT planning to advance participants’ projects. Throughout the sessions, participants applied KT knowledge and principles by working on their own project KT plan using a fillable template provided in curriculum materials. Expectations for active planning included identifying KT goals, determining target audiences, creating key messages, and identifying strategies to support dissemination or implementation initiatives. Each session provided dedicated time as an in-class activity to work through these planning steps. Application of knowledge was demonstrated by participants sharing these completed KT plans in a “showcase activity”; Centre for Leadership Co-Leads and other senior hospital staff engaged in the projects were also invited to this forum. A live KT simulation (designed to encourage participants to synthesize and apply their KT learnings in a realistic situation) offered participants mock opportunities to practice a “KT consult” with colleagues seeking KT guidance [29].

#### Step 6: evaluation and feedback

As part of evaluative activities, data from three sources were gathered to support triangulation of findings: pre/post-curriculum surveys, short post-curriculum interviews, and post-session satisfaction surveys [20]. Post-curriculum assessment was within 2–4 weeks of KTFN completion capturing application of KT knowledge and skills during the project window. Appropriate to the sample size, descriptive analyses were conducted. Likert scale responses were analyzed using frequency distributions. All data, including open-ended survey responses and interview data were organized and coded according to levels 1 and 2: reaction and learning of the New World Kirkpatrick Model [19], as previously outlined. These codes are presented in parentheses in the results section. Minor curriculum modifications were made annually to continue to enhance program delivery.

### Measures

1. Pre/post-curriculum surveys: Participants completed a survey prior to and after the completion of the curriculum, consisting of 13 questions with 5-point Likert scale and open response options to assess changes in knowledge, skills, attitudes, confidence, and behavior. The surveys were developed based on the “retrospective pre/post rating” technique, intended to capture any “response shift” that can happen as a result of a change in understanding of the dimension being rated, that may impact the criteria by which respondents rate themselves in a traditional post-survey [30].
2. Post-curriculum interviews: Brief interviews were also completed after the conclusion of KTFN to gather detailed perspectives of participant experiences and recommendations for curriculum improvements.
3. Post-session satisfaction surveys: After each KTFN session, feedback was gathered using short anonymous satisfaction surveys (16 questions on a 5-point Likert scale with two open text questions) to assess the caliber of KTFN presenters and guest lecturers, session format, content utility, and delivery.

## Results

### Participants

The program was delivered annually to unique cohorts over a 3-year period, with eight participants enrolled in cohort 1, 12 participants enrolled in cohort 2, and 13 participants in cohort 3. The total sample of 33 participants comprised 19 clinical staff, six non-clinical staff (three managers, two researchers, and one Family Partnerships Specialist), two graduate level trainees and six family advisors. Attrition was very low, with two members in year 2 and one member in year 3 withdrawing for personal reasons; 30 participants completed the full curriculum. Participation in program evaluation activities was voluntary, yielding varied response rates across surveys, sessions, and years of delivery. Response rates were high across the three cohorts: 81.8% (*n* = 27/33) completed the pre-survey; 60% (*n* = 18/30) completed the post-survey; and 93.3% (*n* = 28/30) completed the short post-curriculum interviews. Table 4 offers KTFN cohort and evaluation response rate details.

**Table 4:** KTFN cohort and evaluation response details

Cohort participation	Cohort			
	1	2	3	Total
Curriculum delivery	May 2016–November 2016	May 2017–October 2017	May 2018–October 2018	–
Number of participants	8	12^b^	13^c^	33
Profession/role of participants	Healthcare providers: 7^a^ Researchers: 1	Healthcare providers: 7^a^ Researchers: 1 Family advisors: 3 Trainees: 1	Healthcare providers: 5^a^ Management/administrators: 4 Family advisors: 3 Trainees: 1	–
KTFN evaluation response rates	% (*n*)	% (*n*)	% (*n*)	% (*n*)
Pre-survey responses	100 (8/8)	75 (9/12)	76.9 (10/13)	81.8 (27/33)
Session survey 1	87.5 (7/8)	100 (12/12)	83.3 (10/12^c^)	90.6 (29/32)
Session survey 2	87.5 (7/8)	91.6 (11/12)	75 (9/12)	84.4 (27/32)
Session survey 3	100 (8/8)	70 (7/10)^b^	83.3 (10/12)	83.3 (25/30)
Session survey 4	87.5 (7/8)	60 (6/10)	75 (9/12)	73.3 (22/30)
Session survey 5	87.5 (7/8)	90 (9/10)	91.7 (11/12)	90 (27/30)
Session survey 6	–^d^	100 (10/10)	50 (6/12)	72.2 (16/22)
Post-survey responses	62.5 (5/8)	80 (8/10)	41.7 (5/12)	60 (18/30)
Interviews	100 (8/8)	100 (10/10)	83.3 (10/12)	93.3 (28/30)

^a^Some clinical staff held research appointments offering dedicated time to support this activity

^b^Two participants withdrew from the program for personal reasons resulting in 16.7% attrition

^c^One participant withdrew from the program for personal reasons resulting in 7.7% attrition

^d^Delivery of KTFN for cohort 1 occurred over five sessions

### Evaluative findings

#### New World Kirkpatrick model: level 1—reaction

Applying level 1 of the New World Kirkpatrick Model as a framing tool, data were organized according to how satisfactory (favorable), engaging, and relevant the participants found the training [19].

##### Satisfaction (favorable), engagement, and relevance

Across all three cohorts of post-session data, 99.3% of respondents “agreed or strongly agreed” that all KTFN sessions were relevant, and 95% of respondents “agreed or strongly agreed” that “the guest presenter and the EtC presenter made the subject matter compelling”; see Table 5 for relevance and presenter data details. One respondent stated in their post-session survey that “I truly enjoyed today as it stimulated my ideas and passion further about my project…[it] inspired me about how/who/when I need to roll it out successfully with sensitivity for for where people are at who are to be impacted.”

**Table 5:** Session relevance and presenter details

Likert scale responses	Questions								
	The session was relevant^a^			The guest presenter made the subject matter compelling^a^			The EtC presenter made the subject matter compelling^a^		
	Yr. 1^b^	Yr. 2^c^	Yr. 3^c^	Yr. 1^b^	Yr. 2^d^	Yr. 3^d^	Yr. 1^b^	Yr. 2^d^	Yr. 3^d^
	% (*n*)	% (*n*)	% (*n*)	% (*n*)	% (*n*)	% (*n*)	% (*n*)	% (*n*)	% (*n*)
Strongly agree	86.1 (31/36)	83.6 (46/55)	83.3 (45/54)	75 (27/36)	77.8 (35/45)	69.4 (34/49)	72.2 (26/36)	53.3 (24/45)	69.4 (34/49)
Agree	13.9 (5/36)	16.4 (9/55)	14.8 (8/54)	13.9 (5/36)	20 (9/45)	26.5 (13/49)	25 (9/36)	35.6 (16/45)	30.6 (15/49)
Neither agree nor disagree	0	0	1.9 (1/54)	11.1 (4/36)	2.2 (1/45)	4.1 (2/49)	2.8 (1/36)	11.1 (5/45)	0
Disagree	0	0	0	0	0	0	0	0	0
Strongly disagree	0	0	0	0	0	0	0	0	0

^a^Aggregate across sessions

^b^Five sessions

^c^Six sessions

^d^Five sessions as this question was not asked in session 6, the simulation session

In the interviews, respondents indicated a preference for when guest speakers were closely aligned with the session topic; sessions that were more interactive than didactic; and when concrete, real-life examples were provided. One respondent commented that “the guest speaker allowed me to make connections between session content and practical application (i.e. use of examples) and feedback from others.” Respondents identified the need for more time to engage in discussions with other KTFN colleagues. They also expressed they needed more time to work on their own projects in session, as opportunities for these connections and time to complete their KT plan were limited due to competing work-related priorities.

Respondents indicated that KTFN created the opportunity for KT to become relevant to their funded projects in two ways. First by gradually introducing KT concepts, building their knowledge, and helping them to understand how KT was applicable to their project regardless of its stage of development. Second, KTFN encouraged practical applicability by reserving 1 h of each session for participants to work on their individual KT plans, supporting them to incorporate content learned in real-time. In the post-curriculum survey, 81% (*n* = 17/21) of respondents “agreed” or “strongly agreed” that the KTFN covered sufficient content to complete and execute a KT plan for their project.

Through the interviews, respondents highlighted that KTFN increased their access to and engagement with a variety of staff, students, and family advisors across the hospital. From an organizational perspective, this allowed them to learn about other projects taking place, with one respondent saying, “I liked the diversity of backgrounds in the program; we were able to learn from each other.” Additionally, KTFN created internal networking opportunities that may not have otherwise occurred, “it’s important for people in the organization to meet us and vice versa, this is a good way to increase face to face relationships with folks and create a learning organization.” The individual presentations in the final session of KTFN were highpoints for all three cohorts.

#### New World Kirkpatrick model: level 2—learning

In level 2 of the New World Kirkpatrick Model, evaluation efforts focus on participant learning; this was measured through changes in knowledge, skills, attitude, confidence, and intention to use (commitment) knowledge.

##### Changes in knowledge and skills

All three cohorts identified a positive shift, across pre/post-curriculum collection time points, in their awareness of KT literature and strategies (knowledge). As presented in Table 6, responses changed from “not at all” and “slightly aware” (85.2%, *n* = 23/27) to “moderately aware” and “extremely aware” (81%, *n* = 17/21). In open text responses, respondents described “being surprised” to learn that KT is a field underpinned by models, theories, frameworks, and a supporting body of research literature. Through their heightened awareness, they described a deeper appreciation for the potential complexity of KT planning and long-term nature of KT activities.

**Table 6:** Pre/post survey results from years 1, 2, and 3

Likert Scale Responses	New World Kirk Patrick Model Level	Pre-Survey Question	Post-Survey Question
		% (*n*)	% (*n*)
	Level 2: Reaction Changes in knowledge and skills	I rate my awareness of KT literature and strategies associated with doing KT as…	Compared to before attending KTFN, I would now rate my awareness of KT literature and strategies associated with doing KT as…%
Not at all aware		22.2% (6/27)	0
Slightly aware		63% (17/27)	0
Somewhat aware		14.8% (4/27)	19% (4/21)
Moderately aware		0	76.2% (16/21)
Extremely aware		0	4.8% (1/21)
	Level 2: Reaction Changes in knowledge and skills	Based on my current understanding of KT, I would rate my KT skill level as…	Compared to before attending KTFN, I would now rate my KT skill level as…
Novice		37% (10/27)	4.8% (1/21)
Advanced beginner		48.2% (13/27)	57.1% (12/21)
Competent		11.1% (3/27)	38.1% (8/21)
Proficient		3.7% (1/27)	0
Expert		0	0
	Level 2: Reaction Changes in knowledge and skills	I find creating and using KT strategies…	Compared to before attending KTFN, I find creating and using KT strategies…
Very hard		0	0
Hard		25.9% (7/27)	0
Neither easy nor hard		33.3% (9/27)	57.9% (11/19)
Easy		0	31.6% (6/19)
Very easy		0	0
N/A–I have not used a KT strategy in practice		40.8% (11/27)	10.5% (2/19)
	Level 2: Reaction Changes in attitudes	Using KT to implement and share findings from the seed grant funded project is important to me…	Compared to before attending KTFN, I would now rate using KT to implement and share findings from seed grant funded projects as…
Not at all important		0	0
Slightly important		0	0
Moderately important		3.9% (1/26)	4.8% (1/21)
Very important		61.5% (16/26)	42.8% (9/21)
Extremely important		34.6% (9/26)	52.4% (11/21)
	Level 2: Reaction Changes in confidence	I am confident that I can perform effectively on many different KT tasks in my role for the seed funded project…	Compared to before attending KTFN, I am confident that I can perform effectively on many different KT tasks in my role for the seed funded project…
Strongly disagree		0	0
disagree		3.7% (1/27)	0
Neither agree nor disagree		51.9% (14/27)	19% (4/21)
Agree		40.7% (11/27)	81% (17/21)
Strongly agree		3.7% (1/27)	0
	Level 2: Reaction Intention to use KT information	I have used a KT strategy to move evidence into practice...	Since attending KTFN, I have used a KT strategy to move evidence…
Yes		45.8% (11/24)	52.4% (11/21)
No		45.8% (11/24)	9.5% (2/21)
Unsure		8.4% (2/24)	0
Not yet - but I plan to for a future project^a^		–	38.1% (8/21)
Not yet - I do not intend to in future projects^a^		–	0

^a^Likert scale responses were available in the post-survey question only

After attending KTFN, respondents reported positive shifts in KT skills, stating that the program provided them with the necessary skills to use beyond their project. When asked to rank their KT skill level after attending KTFN, participants identified themselves as “competent” or an “advanced beginner” (95.2%, *n* = 20/21) versus 59.3% (*n* = 16/27) in the same categories prior to KTFN (see Table 6).

Respondents further identified that creating and using KT strategies (skills) was no longer viewed as “hard” (*n* = 0), but rather “neither easy nor hard” (57.9%, *n* = 11/19) or “easy” (31.6%, *n* = 6/219) (see Table 6). This was facilitated by having a greater understanding of the types of KT strategies to draw from; recognizing the importance of linking the strategy to the KT goal and needs of the target audience; and having access to resources among learners. One respondent commented that “the biggest change in my thinking has been to incorporate KT from the start of a project and the value of stakeholder partnership.” With another stating that, “KTFN is a hallmark of a learning organization.” Respondents did note that time to plan and conduct KT activities continued to be the biggest barriers and needs to enable future KT work.

##### Change in attitudes

When prompted to respond to the following statement, “using KT to implement and share findings from Centres for Leadership projects is important to me,” responses did not shift greatly across the pre/post collection time points. High value (attitudes) was already placed on KT prior to attending KTFN: pre-KTFN, 96.1% (*n* = 25/26) selected “very or extremely important” and post KTFN, this number remained constant at 95% (*n* = 20/21) (see Table 6). A shift was noted in the timing of KT activities, however. Prior to participating, respondents identified that they understood KT activities as occurring at the end of a project, primarily through end-of-grant (eKT) dissemination activities such as manuscripts and conferences. After KTFN, participants described that they now understood KT activities as a series of iKT processes that occur at the beginning of the project and not as a project “add-on”; further commenting that “before I only thought about eKT, now I think about iKT as soon as an idea is generated, I’m more explicit….”

Generally, respondents developed a heightened awareness of stakeholder engagement and agreed that involving stakeholders in the initial phases of project development was important. KT-oriented thinking shifted from considering KT as “one activity” and moving toward understanding it as a “collection of activities” and strategies that vary across projects. Participants highlighted a better connection with the in-house KT team after attending KTFN, commenting, “I think now I have a beginner's framework and a working relationship with the [EtC] team for consultation. Certainly, I’m beginning to understand what I don't understand, where I can find the information, and who might be able to help me refine ideas.”

##### Change in confidence

In the post-curriculum survey, respondents identified that the KTFN curriculum increased their confidence to effectively perform different KT tasks as part of their role on their project (see Table 6). Confidence was also captured in post-session surveys following the KT simulation (added in year 2 of curriculum delivery); 100% (*n* = 17/17) of respondents indicated that it gave them the confidence to apply KT concepts in practice, commenting that it helped them to “practice the [KT] language and how to talk to others,” and become a KT champion for their colleagues.

##### Intention to use KT information (commitment)

Prior to participating in KTFN, 11 out of 24 (45.8%) of respondents had experience using KT strategies and executing activities to put evidence into practice; notably, these were primarily dissemination focused activities (i.e., conference presentations, publishing, media outlets). Since attending KTFN, 90.5% (*n* = 19/21) of respondents have or plan to use a KT strategy (commitment) to move evidence into practice for their project (see Table 6). The simulation activity in session 6 was again noted as valuable to participants to help them understand “their role as a future KT champion” and how to handle potential “KT push back” from colleagues on future projects.

### Curriculum improvements

Course content was reviewed annually utilizing the evaluative feedback and new literature in the KT field, and modified to reflect user needs. Modifications included removing homework after year 1; increasing the variety of interactive activities throughout the sessions; enlisting new guest speakers to better reflect session topics, adding yearly KT “knowledge burst” emails, and shifting relative emphasis of practical and theoretical aspects of the course.

## Discussion

The Knowledge Translation Facilitator Network was designed to address an organizational need for enhanced KT competencies among staff. The results demonstrate that KTFN shows progress toward achieving this goal. Overall, KTFN was met with a high degree of satisfaction in curriculum delivery and overall perceived satisfaction. Though literature around KT competency has grown since the initial inception of this curriculum, many of KTFN’s competency goals parallel Mallidou et al.’s [9] recently formalized conceptualization of KT competencies and were similarly categorized into the areas of knowledge, skills, and attitude. More specifically, KTFN focused on KT knowledge, which fostered a shift in learners’ self-perceptions of being a KT “novice” to greater perceptions of confidence, with many moving to the level of an “advanced beginner” in their awareness and understanding of KT practice. Further to skill building, participation resulted in an enhanced appreciation of the importance of KT as part of their day-to-day work, as well as confidence in their ability to execute KT activities. Findings in this study align with existing literature on training initiatives focused on KT competency building where participants experienced positive changes in KT knowledge, skills, and attitudes [12, 13].

The delivery structure of KTFN was identified as a key element of its success. As suggested by Holmes et al. [31], creative, practical, and workshop-based approaches are better suited to meet KT training needs than didactic training alone. In this study, content experts and facilitators (e.g., KT specialist, knowledge brokers, and guest lecturers) helped bring the material to life through a pragmatic and context-specific lens using educational strategies such as KT planning exercises, think-pair-share, and case-scenarios. Simulation was also integrated into KTFN as previous literature demonstrates that it can drive knowledge uptake and support skill development within healthcare [32]. We found that simulation supported participant confidence to apply acquired KT knowledge and concepts, similar to other studies that examined the benefits of using this strategy to facilitate KT competency [33].

Furthermore, the requirement of KTFN participants to be engaged in an actively funded project and dedicated time within sessions to work on applying session content in real-time to these projects was an essential curriculum component. This is in line with other training initiatives that highlight the importance of facilitating connections between curriculum content and participants’ projects [12, 13]. An important caveat to note is that some participants found this challenging as their new knowledge led them to reconsider early project design decisions. In some cases, projects could be modified but in others, this was not viable, especially in the case of missed opportunities for stakeholder engagement at project onset, a fundamental aspect of KT. This learning further supports the need for organizational KT competency building to strengthen internal strategic projects moving forward.

KT capacity building within an organization is complex as there are dynamic and interrelated forces at play, including individual and contextual factors. From an organizational perspective, KT capacity can be supported by creating opportunities for its members to interact with KT theories, models, and frameworks. Promoting a culture of shared values and attitudes, enabling engagement in shared practices, and fostering the exchange of ideas and learnings among organizational members is also instrumental [4, 5, 14]. We found that KTFN contributed to these important mechanisms. Participants were oriented to and reflected on foundational KT knowledge (e.g., theories, models, frameworks, principles, strategies) and expressed intention to apply this information.

Additionally, the purposive selection of educational strategies that focused on articulating how KT (i) can be enacted successfully within the internal context and (ii) enables broader organizational goal attainment reflects this organizational capacity building approach. Bringing together a small cohort of learners representing healthcare providers, researchers, trainees, and family advisors allowed various perspectives and experiences to be shared and connections among related work identified. More specifically, participants were able to collaborate with organizational members outside of their typical teams, learn about other initiatives across the organization, and share experiences, challenges, and solutions related to their projects. KTFN also afforded increased interaction between EtC and other members of the organization. Participants reported increased intent to pursue more regular consultation with the in-house KT team moving forward with projects. This form of sustained expertise seeking and/or mentorship from the EtC team is purported as another potential mechanism for facilitating organizational capacity development [4].

Some unanticipated benefits of KTFN participation were also observed. Respondents also valued the opportunity to practice presentation skills in showcasing their projects. Participating in KTFN helped participants appreciate how they were already engaging in eKT activities in a variety of ways but previously did not recognize it as such. They also gained a broader understanding of iKT. This kind of realization, combined with enhanced knowledge and awareness of resources to support KT planning, implementation, and evaluation helped respondents take greater ownership in directing KT aspects of their current projects.

This evaluation does have some limitations. This was a relatively small cohort and though response rates were acceptable, participants may have been biased toward positive responses and there were few non-respondents. Participants were also a highly motivated group of learners intent on growing their competencies. Similar to the findings of Carlfjord et al. [34] in their evaluation of an annual course, participants may be “self-selected” and thus autonomously driven among a group with shared interests, leading to positive experiences. Data collected focused on levels 1 and 2 of the Kirkpatrick Model; longer-term evaluative activities could explore impact longitudinally to determine the extent to which learnings were integrated into future states of participant existing projects as well as new projects. While these data were used in annual accountability reporting and to support requests for renewed curriculum funding for ongoing organizational support, more in-depth follow-up interviews with Centre for Leadership Co-Leads may also yield insightful perspectives on tangible shifts in KT practice.

## Conclusion

Investing in individual KT competency building is an important foundational step in institutionalizing KT within an organization. KTFN facilitated individual change and helped KT gain further momentum at an organizational level. Though we are moving in the right direction, the organization has not reached a point where KT practices are fully integrated and continued focus and energy is needed. Ongoing efforts include engaging the KTFN champions through monthly alumni email blasts to share seminal articles and resources on a given KT topic, hosting annual KT booster workshops for new knowledge and skill development, as well as opportunities to consolidate existing skills. With these ongoing efforts, we aim to continue to expand KT knowledge, skills, and practice across the organization to enhance the lives of children, youth, families, and staff at Holland Bloorview.

## Supplementary information

**Additional file 1.** The TIDieR (Template for Intervention Description and Replication) Checklist*.

## Data Availability

Not applicable.

## Abbreviations

KT: Knowledge translation
KTFN: Knowledge Translation Facilitator Network
EtC: Evidence to care
eKT: End-of-grant KT
iKT: Integrated KT
